# Environmental regulation of the chloride transporter KCC2: switching inflammation off to switch the GABA on?

**DOI:** 10.1038/s41398-020-01027-6

**Published:** 2020-10-15

**Authors:** Davide Pozzi, Marco Rasile, Irene Corradini, Michela Matteoli

**Affiliations:** 1 Humanitas University, Department of Biomedical Science, Via Rita Levi Montalcini 4, Pieve Emanuele, MI Italy; 2 Humanitas Clinical and Research Center — IRCCS, via Manzoni 56, Rozzano, MI Italy; 3 CNR Institute of Neuroscience, Milan, Italy

**Keywords:** Molecular neuroscience, Physiology

## Abstract

Chloride homeostasis, the main determinant factor for the dynamic tuning of GABAergic inhibition during development, has emerged as a key element altered in a wide variety of brain disorders. Accordingly, developmental disorders such as schizophrenia, Autism Spectrum Disorder, Down syndrome, epilepsy, and tuberous sclerosis complex (TSC) have been associated with alterations in the expression of genes codifying for either of the two cotransporters involved in the excitatory-to-inhibitory GABA switch, KCC2 and NKCC1. These alterations can result from environmental insults, including prenatal stress and maternal separation which share, as common molecular denominator, the elevation of pro-inflammatory cytokines. In this review we report and systemize recent research articles indicating that different perinatal environmental perturbations affect the expression of chloride transporters, delaying the developmental switch of GABA signaling, and that inflammatory cytokines, in particular interleukin 1β, may represent a key causal factor for this phenomenon. Based on literature data, we provide therefore a unifying conceptual framework, linking environmental hits with the excitatory-to-inhibitory GABA switch in the context of brain developmental disorders.

## Introduction

Besides genetics, individual susceptibility to psychiatric disorders is known to be influenced by environmental factors, where the term “environmental” encompasses all those factors that are not genetically inherited. The prenatal window and the early phases of post-natal development represent critical periods, when environmental insults may have long-lasting consequences on the brain developmental trajectories^1–4^. Among these insults, stress and inflammation are two recognized risk factors for a wide spectrum of brain disorders, which may emerge either soon after birth or later in postnatal development^5–7^. Since the first indications of the long-lasting effects of stress occurring during pregnancy in the development of behavioral disturbances^8^, evidence is progressively accumulating that prenatal stress may influence birth outcomes and offspring neurodevelopment. In particular, maternal prenatal stress, regardless of whether psychological (e.g., infections or maternal restraints) or physical (e.g. cardiovascular abnormalities), increases the risk of preterm birth^9,10^ and psychiatric conditions such as depression and anxiety^11^, schizophrenia^12^, personality disorders^13^, autism, and ADHD^14^.

In the complex set of intertwined processes found to be defective in several neurodevelopmental disorders, including altered spine morphology and function^15^ and uneven synaptic maintenance/dendritic complexity^16^, the unbalanced excitatory/inhibitory (E/I) transmission^17,18^ and the altered GABAergic function^19,20^ represent highly investigated potential pathogenic mechanisms, possibly accounting for the lack of a balanced and flexible neuronal network typical of neuropsychiatric patients^21^. In the developmental sequence of events governing the inhibitory neurotransmission maturation, a central role is played by the so-called excitatory-to-inhibitory GABA switch. Discovered almost three decades ago, the GABA switch relies on the occurrence, at early developmental stages, of elevated intracellular chloride levels which progressively reduce, giving rise to an excitatory-to-inhibitory shift of GABA action. A temporal deviation from this switch leads to the persistence of immature GABAergic features in the adult brain, which perturb the operation of well-developed functional networks^22^. According to its functional importance during development, GABA is implicated in a number of neurodevelopmental disorders such as Autism Spectrum Disorders (ASD)^23^, Fragile X^24^, Rett syndrome^25,26^, Down syndrome^27^, and schizophrenia^28^. In this review, we discuss experimental evidence indicating that developmental GABA switch is largely influenced by environmental stimuli, thus possibly representing a central link that translates early-life environmental hits into behavioral changes in the adult.

## Neuronal chloride homeostasis: a key factor in brain maturation

The discovery of the dichotomic action of GABAergic neurotransmission dates back to over 30 years ago^29,30^. The inhibitory action of GABA in mature neurons relies on its ability to open chloride (Cl^−^)-permeable GABA_A_ receptor channels that allow Cl^−^ influx and hyperpolarization. However, at early stages of development, when intracellular Cl^−^ concentrations are high and Cl^−^ equilibrium potential is positive, compared to the resting membrane potential, GABAergic neurotransmission results in Cl^−^efflux and membrane depolarization. The homeostatic regulation of intracellular ionic composition, which ends up determining the polarity of GABA action in neurons, is ensured by a variety of ion-transporters localized at the cell surface (Table 1). Cation chloride co-transporters (CCCs) represent a group of ion-transporters involved in the control of electrolyte homeostasis through the coupling of cations transport with Cl^−^ in opposite directions^31^. The solute carrier 12 (SLC12) family of CCCs includes four potassium (K)-Cl co-transporters (KCCs) isoforms, named KCC1, KCC2, KCC3, and KCC4, encoded by distinct SLC12 genes (SLC12A4-7 respectively)^32^ and two sodium (Na)-K-Cl cotransporters, namely NKCC1 and NKCC2, encoded by SLC12A2 and SLC12A1 genes, respectively. In particular, NKCC cotransporters drive Cl^-^ uptake fueled by Na^+^^33^, while Cl^-^ extrusion is fueled by K^+^ through the KCC cotransporters^34–38^. All these transporters derive their energy from the Na^+^ and K^+^ gradient generated by Na-K-ATPase. NKCC2 exhibits kidney-specific expression and functions in renal salt reabsorption, whereas NKCC1 plays a key role in regulation of salt secretion and cell volume in epithelial cells and is critical for the appropriate response to GABA in neuronal cells. Both NKCC isoforms are inhibited by 5-sulfamoylbenzoic acid loop diuretics, including bumetanide. Concerning KCCs, KCC1 was found to be ubiquitously expressed by northern blot analysis^34^, while KCC2, initially identified only in the brain^37^, has been recently found also in chicken myocytes^39^; KCC3 and KCC4 are expressed in both the central and peripheral nervous systems (PNS)^40–42^, heart, skeletal muscle, and kidney^35,36^ (Table 1). KCC2 exhibits two isoforms, named KCC2a and KCC2b, which differ in their most N-terminal part^43^, and is barely detectable in the PNS and non-neuronal cell types as glial cells^37,44–46^. Unlike the other KCC-family members -whose levels remain stable over time- KCC2 expression follows a robust upregulation during brain development^47^, a process indispensable for a correct neuronal differentiation and functioning^48^. In particular, increases occur in the cortical expression of KCC2b isoform over KCC2a during late postnatal development, while the two isoforms display nearly comparable changes in the brain stem and spinal cord during prenatal and early postnatal stages^43^. KCC2a expression is therefore barely expressed in, or even lacking from, most parts of the adult cortex, hippocampus, thalamus, and cerebellar cortex, whereas both isoforms are detectable in the developing and mature hypothalamus, brainstem (except for the brainstem auditory system that lacks KCC2a immunoreactivity), and spinal cord^49^ (Table 1). This molecular dynamic has been also reported in other species, from reptile to humans, indicating that the developmental GABA switch is an evolutionary conserved process^50,51^. The progressive increase of KCC2b activity during neuronal development, together with the progressive reduction of NKCC1, causes the lowering of intracellular chloride levels and set the driving force and the reversal potential of the anion currents^31^. This allows the physiological transition from the excitatory to the inhibitory action of GABA^51–56^. Notably, the depolarizing GABA-A transmission is implicated in numerous developmental processes, including neural stem cell proliferation^57^, cell migration^58^, neurite outgrowth^59^, synapse formation and circuit refinement^59–61^ as well as shaping excitatory connectivity during neural circuit development^62^. The dual role played by GABAergic transmission represents therefore one of the fundamental pillars of brain development, ensuring a proper brain wiring and functioning at adult stages.

**Table 1:** Localization and developmental regulation of chloride transporter KCC and NKCC isoforms.

	

This cartoon was adapted and modified from Servier Medical Art (http://smart.servier.com/).

## Modulating GABA switch through KCC2 regulation by hormonal and soluble factors: implications for neurodevelopmental diseases

The molecular processes controlling KCC2 expression are subjected to multiple regulatory mechanisms relying on both transcriptional and post-transcriptional pathways. At transcriptional level, KCC2 gene is modulated via the coordinated activity of several transcription factors, such as RE1-Silencing Transcription factor (REST)^63^, a neuronal gene repressor that plays a crucial role in neuronal differentiation. REST activity depends on its interaction with other cofactors, including CoREST and MeCP2, thereby forming a complex transcriptional machinery which ultimately regulates the expression of many neuronal-specific genes. Interestingly, the involvement of the transcription factor methyl-CpG-binding protein 2 (MeCP2) in the control of KCC2 expression has been recently reported, supporting the interplay between REST and MeCP2 in this process^64,65^.

Several soluble factors are also known to influence the process of GABA switch. The first pioneer study^66^, subsequently corroborated by a more recent research article^67^, proposed that GABA signaling *per se* represents the fundamental extracellular cue that allows the occurrence of the switch and the progressive upregulation of KCC2 transporter. The GABA switch process is also promoted by trophic factors including the neurotrophic factor BDNF^68–70^, which exerts a facilitatory effect on the expression of KCC2 mRNA and protein, during development. The effect of BDNF on KCC2 protein expression occurs via extracellular signal-regulated kinase 1/2 (ERK1/2)-dependent upregulation of the transcription factor early growth response 4 (Egr4) and via Egr4-dependent activation of the KCC2b promoter^70^.

Hormonal factors also play a central role in GABA switch modulation. For instance, estradiol downregulates KCC2 mRNA^71^, while testosterone and dihydrotestosterone upregulate KCC2 mRNA^72^. Similarly, the thyroid hormone triiodothyronine (T3) enhances the expression of KCC2 protein, thus accelerating the developmental shift of GABA action from depolarizing to hyperpolarizing^73^. A special attention has been paid to oxytocin, which exerts a crucial role in the control of GABA developmental shift during labor. Shortly before delivery, the surge of maternal oxytocin triggers, in the offspring brain, a transient switch of the GABA response from excitatory to inhibitory^74–76^ that is crucial for the entire subsequent neurodevelopmental process. Oxytocin positively promotes the plasmamembrane insertion of KCC2 through a change of the phosphorylation state of the transporter^77^, highlighting the relevance of post-translational mechanisms in the transporter regulation. Among the kinases that control the phosphorylation of KCC2, thus regulating the transporter activity and/or its cell surface stability (for review see, ^78,79^), PKC is mainly involved in the phosphorylation of several serine residues, cytosolic c-Src kinase^80^ and BDNF-dependent TrkB receptor kinase target tyrosine residues of KCC2^68,81,82^ whilst the WNK (with-no-lysine[K]) family of serine-threonine kinases together with SPAK, Ste20p-related proline/alanine-rich kinase modulate the phosphorylation level at threonine residues. Regarding the latter, WNK/SPAK-kinase activity is reduced during neuronal development, leading to a substantial dephosphorylation of threonine 906 and 1007 and a consequent increased of KCC2 activity^83^.

The key role of hormonal factors in the regulation of KCC2 is in line with the reported sex-biased functional maturation of GABA inhibitory signaling, which occurs, in a brain region-specific manner, earlier in females than in males^71,84–86^. While the male hormone testosterone or its metabolites at early post-natal period appear to be crucial for GABA switch in the substantia nigra (SNR) in both sexes, via the upregulation of KCC2 mRNA, the female hormone estradiol downregulates KCC2 mRNA in males but not in females^72^. In the hippocampus and entorhinal cortex of neonatal rats, KCC2 remains consistently higher in females compared to males especially in the second post-natal week, when NKCC1 peaks are detectable only in males^87^. Notably, however, hypothalamic cells obtained from embryos before the first testosterone peak, already display sex-dimorphism in their response to the GABAA-receptor agonist muscimol, indicating that differences in GABA switch timing can also occur independently of sex hormones exposure^88^.

The earlier functional maturation of GABAA inhibitory signaling in females^71^ could contribute in making the male GABAergic system less resilient to early environmental insults^89^, with possible, important implications for brain disorders, such as epilepsy and neurodevelopmental diseases. For example, reduction of KCC2 expression and function appears to contribute to the development of epileptic seizures^52^. Interestingly, stressors in early life accelerate epileptogenesis in a sexually dimorphic manner, with males showing a higher susceptibility than females, both in rodents^90^ and in humans^91^. It is important to note that neurodevelopmental diseases, such as ASD and schizophrenia -which display a sex-biased incidence, with ASD affecting males 3 times and schizophrenia 1.4 times more than females^92–94^- are characterized by defects in GABAergic maturation. Alterations of the GABA switch has been recently detected in neurodevelopmental disorders associated or not associated with autism-like behaviors, including Fragile-X^95^ and Rett syndrome^26,65,96,97^. Neurons from a mouse model of Rett syndrome (lacking Mecp2) or derived from induced pluripotent stem cells (iPSCs) of patients with Rett syndrome exhibit a more depolarizing GABA signaling^26^ that could be rescued by either KCC2 exogenous expression^65^ or pharmacological drugs enhancing KCC2 activity^97^. Consistently, reduced KCC2 expression has been observed in post-mortem brain tissues from Rett syndrome patients^98^. Similar phenotypes have been found in Fragile-X syndrome mouse models, where the developmental shift of GABA signaling was found to be impaired owing to KCC2^75,76^ or NKCC1^95^ deregulation. Moreover, in Down syndrome models, the GABA signaling in the adult brain is excitatory instead than inhibitory^53^. Also, impairment of the inhibitory strength of GABAergic neurotransmission consequent to altered KCC2 Thr906/Thr1007 phosphorylation, results in increased seizure susceptibility, altered ultra-sonic vocalization, and social interaction deficits, that are typical of ASD^99^. Finally, decreased expression of KCC2 or increased NKCC1/KCC2 ratio was observed in human cortical brain samples from Dravet syndrome patients^100^ and from TSC patients, respectively^101^.

Furthermore, a differential expression of KCC2 transcripts has been reported in schizophrenic patients^50^, who display a hippocampal NKCC1/KCC2 ratio that is generally higher relative to healthy subjects^50^. Also, KCC2 truncated splice variants are distinctly expressed in schizophrenic patients compared to healthy controls, likely reflecting the altered GABA physiology associated with the disease^102^. Finally, two rare functionally impairing variants in the KCC2 C-terminal regulatory domain have been detected in human ASD (R952H and R1049C) and schizophrenia (R952H). These variants significantly decrease KCC2-mediated Cl− extrusion capacity in neurons, reducing the transporter plasmalemmal expression (R952H) or lowering the intrinsic activity of transporters at the cell surface (R1049C)^103^.

These data clearly indicate that altered KCC2 regulation impacts the GABAergic developmental sequence in vivo, representing a risk factor for the emergence of neurological pathology, such as epilepsy, ASD, schizophrenia. The causal involvement of NKCC1 and KCC2 sexual dimorphism in these disorders is an emerging possibility that deserves further investigation.

## The possible role of inflammatory pathways in KCC2 regulation

The impact of environmental stimuli in the regulation of KCC2 expression, and therefore in the timing of GABA switch, has been demonstrated in protocols of prenatal or early life stress, where an altered GABAergic signaling, leading to an E/I imbalance, occurs in the offspring (Table 2). Pregnant mice subjected to immobilization paradigms at late stages of pregnancy (GD15) give birth to offspring characterized by downregulation of KCC2 expression associated with changes of specific GABA receptor subunits^104^, reduction of several GABAergic markers (e.g., GABA and GAD67)^105^, and significant delayed migration of inhibitory progenitors^106^ (Table 2). Stressful conditions occurring at early post-developmental stages also impact GABAergic signaling. Indeed, animal models of maternal separation, based on daily rounds of pup separation from the mother lasting from the first postnatal day up to 2 weeks, display longer latency to play and decreased social play behavior^107^, and anxiety-like states^108^, associated with KCC2 deregulation delayed GABA developmental switch and excitatory GABA action^109,110^. However, it should be noted that in a different experimental setting based on 6 h daily separation from the dam at P4 for just 3 days, KCC2 immunoreactivity was found to be increased only in males and not females, indicating a gender-specific effect of early post-natal stress on KCC2 expression^111^. Mice subjected to acute (30 min) or chronic restraint stress (30 min/day for 14 consecutive days) display in the hippocampus^112^ or in the paraventricular nucleus of the hypothalamus (PVN)^113^ the dephosphorylation of KCC2 residue S940, which regulates KCC2 cell surface expression and function, accompanied by increased susceptibility to seizures^112,113^ (Table 2). Consistently, induction of repeated stress by forced water administration is associated with decreased KCC2 and increased NKCC1 membrane expression in granular and pyramidal cells in the hippocampus^114^. Interestingly, acute restraint stress performed in young animals is associated with loss of synaptic inhibition coupled with reduced KCC2 activity in the PVN^115^, a key cellular component of the hypothalamic-pituitary stress axis (HPA), leading to a reduced inhibitory constraint at HPA thus participating in the physiological control of stress hormones release^115^ (Table 2). Despite these lines of evidence, the research for cellular defects and signaling pathways activated by early life stress did not provide a clear molecular link yet between these early stressful events and KCC2 deregulation.

**Table 2:** Summary of literature evidence for the regulation of KCC2 expression upon paradigms of environmental challenges performed at either prenatal or postnatal developmental stages.

	Age	Environmental challenge	Duration	Effect	Brain region	Species	Sex	Reference
Prenatal	GD9	Maternal immune activation (PolyI:C)	Acute	↓KCC2 (PND20-90), ↑ NKCC1 (E17)	Cortex	Mouse	Mix	[64]
	GD14-21	Maternal restraint stress	Chronic (7 days)	↓KCC2 (PND22-40),↑KCC2 transient (PND21), ↓NKCCl transient (PND14)	Hippocampus	Rat	NS	[104]
	GD15	Maternal restraint stress or betamethasone i.p. (2x0,4mg/kg)	Acute	↓KCC2 (PND7-15)	Cortex	Rat	Mix	[105]
	GD16-PND14	Environmental enrichment	Chronic (20days)	↑KCC2 (PND2)	Forebrain and hippocampus	Mouse	Both	[148]
Postnatal	PND1-21	Maternal separation	Chronic (21days)	↓KCC2 (PND35–38), = NKCC1	Hippocampus	Mouse	Male	[109]
	PND2-14	Maternal separation	Chronic (13days)	↑KCC2 (PND40), ↑NKCCl (PND14)	Hippocampus	Rat	Mix	[110]
	PND4-6	Maternal separation plus saline injection	Chronic (3 days)	↑KCC2 (PND10 - males only)	Hippocampus	Rat	Both	[111]
	PND21-28	Restraint stress	Acute	↓KCC2 activity	Paraventricular nucleus	Rat	Male	[115]
	8–10 weeks	Restraint stress	Chronic (14days)	↓KCC2, ↓pKCC2ser940	Hippocampus	Mouse	Male	[112]
	8–11 weeks	Forced administration of water	Chronic (21days)	↓KCC2, ↓pKCC2ser940, ↑NKCCl	Hippocampus	Mouse	Female	[114]
	12 weeks	Restraint stress	Acute	↓KCC2, ↓pKCC2ser940	Paraventricular nucleus	Mouse	Male	[113]
	Adult	Exercise after spinal cord transection at T12	Chronic (7-28days)	KCC2/NKCC1 ratio restored	Spinal cord	Rat	Female	[145]
	Adult	Exercise after spinal cord transection at T12	Chronic (23days)	KCC2/NKCC1 ratio restored	Spinal cord	Rat	Female	[144]

GD (gestational day); PND (postnatal day); NS (not specified). Arrows indicate either increased (↑) or decreased (↓) expression, while (=) indicate that no changes were found. Only KCC2 and NKCC1 variations, when indicated, have been reported.

Nonetheless, it is interesting to note that chronic stress induced by maternal separation engaged inflammatory pathways in neurons. In mice subjected to maternal deprivation, increased levels of the interleukin-1 receptor (IL-1R) are detectable specifically at synapses in the male hippocampus, together with enhanced interactions with the GluN2B subunit of NMDARs^116^. Similarly, rats subjected to maternal separation for 3 h/day from post-natal day 1 to 14 and examined at post-natal day 15, displayed higher hippocampal levels of interleukin-1beta (IL-1β) mRNA^117^. The classical restraint stress paradigm during pregnancy (from E10 to E16 for 2 h/day) results in female offspring with elevated IL-1β levels in both placenta and brains^118^. Notably, early and prenatal stress are associated with inflammation also in humans. Social and psychological adversities in children are associated with high CRP (C-reative protein) levels^119–121^ (for a specific review see^122^).

A direct demonstration that a proinflammatory environment reduces the transcription of KCC2, thus delaying the excitatory to inhibitory GABA switch, has been recently provided^64^. Using a protocol of prenatal immune activation employing the synthetic analog of double-stranded RNA polyI:C, the enhanced binding of the transcription factors MeCP2 and REST to the KCC2 gene promoter region was demonstrated in the offspring brain. The consequent reduction of KCC2 protein was accompanied by increased susceptibility to kainate-induced seizures in the adult mouse offspring^64^. This was the first evidence highlighting an effect of maternal immune activation on the GABA developmental shift. A subsequent study^123^ reported that PolyI:C injection at later gestational day, GD12.5, abolished the GABA switch in the offspring, leading to defects that were detectable already at birth. When IL-1R knockout mice, which display silenced IL-1β pathway, were implanted in wild-type dams and prenatally exposed to poly I:C, no reduction of KCC2 could be demonstrated, pointing to a direct involvement of IL-1β in down regulating KCC2 transcription^64^. Accordingly, exposure of cultured neurons to IL-1β resulted in higher chloride intracellular concentrations. Therefore, inflammation-derived molecules produced during fetal development lead to a deregulation of the GABA developmental shift, which manifests at early postnatal stages and persists in the long-term period.

Interestingly, elevation of proinflammatory cytokines has been demonstrated in the pathological contexts where reduction of KCC2 has been found to be involved. IL-1β is recognized to contribute to epilepsy development^124^, and the therapeutic effect of the IL-1β antagonist, Anakinra, has been reported in the relapsing chronic phase of febrile infection–related epilepsy syndrome^125^, as well as in the super-refractory status epilepticus and febrile infection-related epilepsy syndrome^126^. Along the same line, a robust increase in IL-1β and IL-1 receptor were detected in post-traumatic epilepsy in children, and IL-1Ra treatment reduced seizure susceptibility 2 weeks after traumatic brain injury compared to vehicle^127^. In ASD, the aberrant expression of cytokines and their signaling intermediaries has been demonstrated in the brain^128,129^. Elevated peripheral IL-1β levels are associated with the ASD diagnosis later in childhood and vary with ASD symptom severity^130,131^. In high-functioning male children with ASD, the plasma levels of IL-1β, IL-1 receptor antagonist, together with IL-5, IL-8, IL-12(p70), IL-13, and IL-17, were fund to be elevated relative to age-matched controls^132^. These elevations may reflect a prenatal immune challenge^130^. Marked elevations of IL-1β in CSF of male patients with first-episode schizophrenia has been described^133^, while the interleukin-1 cluster was found to associate with genetic risk for schizophrenia^134^. Furthermore, polymorphisms of the interleukin-1 gene complex were demonstrated in schizophrenic patients^135^, and a gender-specific association of interleukin 1 receptor antagonist polymorphism with schizophrenia susceptibility has been detected in African male population^136^.

The reports outlined above converge on the evidence that inflammatory mediators, and in particular IL-1β, may be central in the control of KCC2 expression. Of note, both environmentally- or genetically-induced elevations of this proinflammatory cytokines associate with pathological conditions, that frequently manifest in a gender-specific manner, characterized by altered KCC2 expression.

Through which processes do these environmental stimuli induce the described changes? Although the exact molecular mechanisms are far from being defined, few lines of evidence point to the possible occurrence of epigenetic modifications of the *Kcc2* gene. For example, the early exposure to environmental xenobiotics (i.e. Bisphenol) can delay the developmental expression of KCC2, by increasing MeCP2 binding to the *Kcc2* promoter, similarly to what observed in paradigms of prenatal immune activation^64^. Interestingly, the inhibition of DNA methyltransferase or Histone Deacetylase (HDAC) rescues the delay, suggesting the involvement of epigenetic factors in this process. Similarly, persistent inflammatory pain results in KCC2 suppression through HDAC-mediated histone hypoacetylation^137^, while the suppression of HDAC2 upon spinal cord mechanical hyperalgesia restores KCC2 expression and alleviates symptoms^138^. Interestingly, during infections, proinflammatory cytokines support the immune response through chromatin modifiers and epigenetic regulators, which contribute to the altered gene transcription patterns^139^. The possibility of a link among environmental cues, inflammation-derived molecules and epigenetic regulation of genes, including KCC2, is therefore very likely.

## Reducing inflammation to enhance KCC2 expression?

**Fig. 1:**
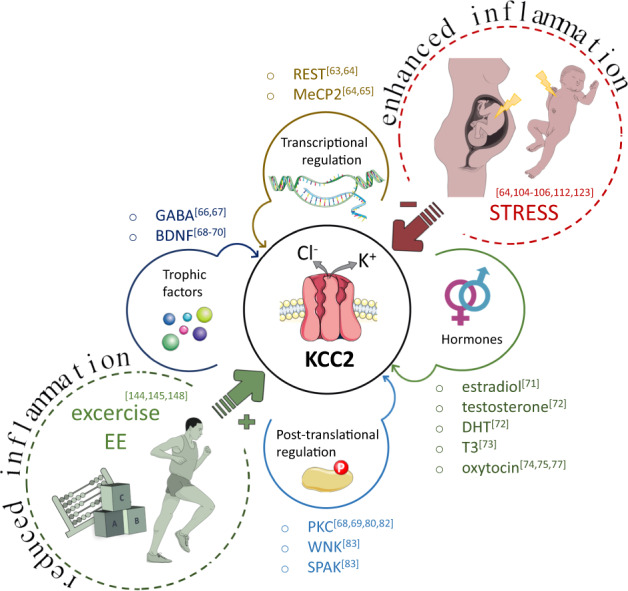
Schematic representation of the agents known to influence developmental GABA switch through KCC2 expression. KCC2 expression levels are regulated through the transcription factors REST and MeCP2, while KCC2 post-translational modifications control the transporter functionality and its insertion into the plasmamembrane (see text for details). Hormones and trophic factors are recognized soluble regulators of KCC2 expression. Stress occurring at either prenatal or early post-natal stages is a main environmental stimulus that reduces KCC2 expression, while exercise/environmental enrichment increase the transporter expression. Stress and exercise/EE likely control KCC2 expression through the modulation of inflammatory pathways. Inflammation, and in particular IL-1β -which is elevated by stress and reduced by exercise/EE-, lowers KCC2 expression levels, by acting through transcription factors or neurotrophic factors. REST (RE1-Silencing Transcription factor); MeCP2 (methyl-CpG-binding protein 2); DHT (dihydrotestosterone); T3 (triiodothyronine); WNK (with-no-lysine[K] family of serine-threonine kinases); SPAK (Ste20p-related proline/alanine-rich kinase). The figures were adapted and modified from Servier Medical Art (http://smart.servier.com/).

The above literature reports open the exciting scenario that inflammation and inflammatory cytokines contribute in controlling the expression of KCC2, a central actor in the developmental sequence at the basis of the inhibitory neurotransmission maturation. As demonstrated, IL-1β could reduce KCC2 expression via enhanced binding of the transcription factors MeCP2 and REST to its gene promoter region^64^. However, other mechanisms may contribute, such as the capacity of the proinflammatory cytokines to influence the release of soluble factors, like hormones, which are known to affect the GABA developmental shift^71^. Of note, immune hits, like stress and inflammation, could amplify the physiological differences of KCC2 detected among sexes, thus differently contributing to alterations of the GABAergic system maturation in males and females (Fig. 1).

These findings could be exploited in the search of paradigms aimed to accelerate the GABA switch under pathological contexts. So far only bumetanide, a FDA-approved loop diuretic that acts by antagonizing NKCC1 and NKCC2, has been tested as a potential chloride homeostasis-restoring drug in the context of several diseases, including neonatal seizures, temporal lobe epilepsy, autism and schizophrenia, in preclinical rodent studies and in off-label clinical studies (for a review see^140^), as well as in adult mouse models of Down syndrome^53^ and DiGeorge syndrome (22q11.2 microdeletion)^141^. The evidence reported above, however, suggest that the acceleration of GABA switch could be achieved also through the exploitation of either pharmacological or not pharmacological strategies aimed at reducing inflammation. Although conceptually consistent, pharmacological approaches aimed at inhibiting cytokine-dependent signaling in the attempt to restore a normal KCC2 expression should be considered with caution. Indeed, it is known that several immune molecules, besides acting as inflammatory mediators, have crucial roles in physiological brain development^142,143^, hence their pharmacological modulation, especially during brain development, might result in untoward effects. Unlike pharmacological-based approach, exercise and environmental enrichment, may bring important contributions as non-invasive strategies aimed to restore GABAergic maturation (Fig. 1). Exercise modulates chloride homeostasis after spinal cord injury, returning toward normal KCC2 levels^144,145^ whose downregulation contribute to the development of spasticity and dysfunctions^82,146^. Similarly, environmental enrichment paradigms -i.e., the addition of social, physical and somatosensory stimulation able to increase synapse formation, plasticity, and neurogenesis^147^- enhance the GABAergic neurotransmission at one week after birth by accelerating the transition of GABA action from excitation to inhibition^148^.

Although the molecular processes by which exercise and environmental enrichment increase KCC2 levels are still to be defined, neurotrophins are likely to play a major role in this process. Exercise is recognized to upregulate expression of BDNF (for a review see^149^), which is a major determinant of KCC2 upregulation. Similarly, IGF-1, that is elevated in the visual cortex upon environmental enrichment, has been found to decrease the ratio between the expression of NKCC1 and KCC2, promoting the developmental switch of GABA polarity from excitation to inhibition^150^. Of note, physical exercise and environmental enrichment are also linked to the ability of preventing and modulating inflammatory conditions. Voluntary wheel-running in mice reduces the expression of key drivers of the cytokine cascade, including IL-1β^151–153^, while environmental enrichment reduces IL-1β and CD68 expression^154^, also modifying microglia toward a resting phenotype^155^. Interestingly, early environmental interventions normalize the immunological dysfunctions produced by prenatal restraint stress, reverting most of immunological alterations and reducing IL-1β levels^156^. Whether this paradigm may also rescue KCC2 expression under the same conditions is still to be defined.

## Conclusions

Besides being genetically defined, KCC2 expression can be regulated by environmental stimuli, such as stress and inflammation (Fig. 1). The recent evidence that the chloride transporter expression is controlled by proinflammatory cytokines, alongside the engagement of inflammatory molecules upon stressful events, open the possibility that these immune molecules might represent the common molecular link between these stimuli and KCC2 alteration. IL-1β, that increases upon infection but also during prenatal stress and maternal deprivation, appears to reduce the transporter expression, delaying the GABA switch and resulting in abnormal brain development. Under this perspective, pharmacological or environmental paradigms aimed to reduce the inflammatory load could represent promising strategies to normalize KCC2 levels. This approach could be possibly attempted even in the context of developmental disorders consequent to prenatal or early postnatal environmental stressors, a medical situation which still completely lacks preventative pharmacological approaches.
